# Evaluation of potential rabies exposure among Japanese international travelers: A retrospective descriptive study

**DOI:** 10.1371/journal.pone.0287838

**Published:** 2023-08-18

**Authors:** Hidetoshi Nomoto, Kei Yamamoto, Satoshi Kutsuna, Yusuke Asai, Yu Kasamatsu, Michinori Shirano, Toshinori Sahara, Fukumi Nakamura, Yukiko Katsuragi, Masaya Yamato, Koh Shinohara, Naoya Sakamoto, Ryota Hase, Taku Ogawa, Atsushi Nagasaka, Nobuyuki Miyata, Norio Ohmagari

**Affiliations:** 1 Disease Control and Prevention Center, National Center for Global Health and Medicine, Tokyo, Japan; 2 Emerging and Reemerging Infectious Diseases, Graduate School of Medicine, Tohoku University, Miyagi, Japan; 3 Department of Infection Control, Graduate School of Medicine, Osaka University, Suita, Osaka, Japan; 4 AMR Clinical Reference Center, Disease Control and Prevention Center, National Center for Global Health and Medicine, Tokyo, Japan; 5 Department of Infectious Diseases, Osaka City General Hospital, Osaka, Japan; 6 Department of Infectious Diseases, Tokyo Metropolitan Health and Hospitals Corporation Ebara Hospital, Tokyo, Japan; 7 Department of General Internal Medicine and Infectious Diseases, Rinku General Medical Center, Osaka, Japan; 8 Department of Infectious Diseases, Kyoto City Hospital, Kyoto, Japan; 9 Department of Clinical Laboratory Medicine, Kyoto University Graduate School of Medicine, Kyoto, Japan; 10 Department of Infectious Diseases, Tokyo Metropolitan Bokutoh Hospital, Tokyo, Japan; 11 Department of Infectious Diseases, Japanese Red Cross Narita Hospital, Chiba, Japan; 12 Center for Infectious Diseases, Nara Medical University, Nara, Japan; 13 Department of Infectious Diseases, Sapporo City General Hospital, Hokkaido, Japan; 14 Department of Infectious Disease, Yokohama Municipal Citizen’s Hospital, Kanagawa, Japan; Universidad Santo Tomas, CHILE

## Abstract

**Background:**

Although Japan has been a rabies-free country for >50 years, a few cases have been reported among people traveling abroad. This study aimed to investigate animal exposure among Japanese travelers using the Japanese Registry for Infectious Diseases from Abroad (J-RIDA).

**Method:**

In this retrospective analysis, we examined Japanese overseas travelers with animal exposure, as included the J-RIDA database, reported from October 1, 2017, to October 31, 2019, with a focus on pre-exposure prophylaxis (PrEP) administration and the animals to which the patients were exposed.

**Results:**

Among the 322 cases included in the analysis, 19 (5.9%) patients received PrEP and 303 did not. The most common purpose of travel was a non-package tour (n = 175, 54.3%). Most trips (n = 213, 66.1%) were to a single country for <2 weeks. Most patients (n = 286, 87.9%) traveled to countries with a rabies risk. The majority of patients with and without PrEP were injured in rabies-risk countries [n = 270 (89.1%) for non-PrEP and n = 16 (84.2%) for PrEP]. Animals associated with injuries included dogs (55.0%), cats (25.5%), and monkeys (15.5%). Most patients were classified as World Health Organization Category II/III for contact with suspected rabid animals (39.5% and 44.1% for categories II and III, respectively) and had exposure within 5 days of travel. Southeast Asia (n = 180, 55.9%) was the most common region in which travelers were exposed to animals.

**Conclusions:**

Japanese overseas travelers had contact with animals that could possibly transmit the rabies virus, even on short trips. Promoting pre-travel consultation and increasing awareness of the potential for rabies exposure are important for prevention of rabies among Japanese international travelers.

## Introduction

Rabies is a lethal zoonotic disease that causes an estimated 59,000 human deaths annually in over 150 countries globally [1]. More than 95% of rabies cases are reported from Asia and Africa due to restricted accessibility to the rabies vaccine, unvaccinated stray animals, and poor populations in rural areas; and children under the age of 15 years are more likely to be affected [2]. Although 99% of rabies cases are dog-related, cats and wild animals, including bats, foxes, raccoons, and skunks, can also transmit rabies [3]. Vaccines are effective in preventing rabies and are recommended for travelers who are at risk of rabies exposure through travel to rabies-endemic countries [4].

Many developed countries and islands, such as Japan, have adequate disease surveillance and have not reported any rabies cases in land animals in recent years [5]. The last rabies cases among humans and domestic animals in Japan were reported in 1954 and 1957, respectively. However, a few cases have been reported among travelers. An imported case was reported in a traveler from Nepal in 1970, and two concurrent imported cases were reported in travelers from the Philippines in 2006 [6–8]. After the reporting of these three imported cases, no cases of rabies were reported in Japan for 14 years. However, on May 22, 2020, a person traveling for work from the Philippines developed rabies [9]. The rapid increase in international travel during recent years has raised concerns about the possibility of imported rabies cases in Japan [10]. Nevertheless, few studies have reported on animal exposure that could transmit rabies among Japanese travelers [9, 11–13].

The purpose of this study was: (1) to study the characteristics of Japanese international travelers based on pre-exposure prophylaxis (PrEP) status; and (2) to examine the actual situations of animal exposures during travel. We utilized data from the Japanese Registry for Infectious Diseases from Abroad (J-RIDA), a multicenter registry for imported infectious diseases in Japan [14], aiming to provide a better understanding of rabies exposure and effective preventive measures among Japanese international travelers.

## Material and methods

### Study design and data collection

A retrospective, descriptive study of animal-related exposure in Japanese travelers was conducted from October 1, 2017, to October 31, 2019, using the J-RIDA database. The J-RIDA case registration system was developed and maintained by the Joint Center for Researchers, Associates, and Clinicians Data Center at the National Center for Global Health and Medicine, Tokyo, Japan (https://www.jcrac.info/redcap/). Data entry to this system began in October 2017. As of December 2019, 16 centers, namely the National Center for Global Health and Medicine, Osaka City General Hospital, Rinku General Medical Center, Tokyo Metropolitan Health and Hospitals Corporation Ebara Hospital, Tokyo Metropolitan Bokutoh Hospital, Japanese Red Cross Narita Hospital, Kyoto City Hospital, Sapporo City General Hospital, Nara Medical University Hospital, Yokohama Municipal Citizen’s Hospital, Kagawa Prefectural Central Hospital, Toyama University Hospital, Nagoya City East Medical Center, Japanese Red Cross Nagoya Daini Hospital, Kyushu University Hospital, and Aomori Prefectural Central Hospital, had registered as collaborative facilities. Patients’ data, including demographics, travel history, pre-travel vaccination and consultation, country of exposure, and clinical evaluation, were collected. Participating medical institutions in J-RIDA registered all cases determined to be travel-related in the registry. In this study, data were extracted for cases among Japanese patients who visited these medical institutions because of animal exposure acquired while traveling in other countries.

The data retrieved from the database included the following: (i) demographics, including age, sex, travel details, availability of pre-travel consultation, and outcome; and (ii) animal-related exposure stratified by the type of animal. Animal exposure was classified into three categories according to the existing World Health Organization (WHO) definitions [15] as follows: Category I, touching, feeding, or licks on intact skin; Category II, nibbling of uncovered skin or minor scratches without bleeding; Category III, scratch or bite wounds that penetrate the skin, or contamination of mucous membranes by saliva. Category II and III exposures are regarded as wounds at risk of developing rabies. To determine whether the country where a patient had the animal exposure was a rabies-risk country, we used the country-specific rabies-risk assessment published by the US Centers for Disease Control and Prevention [16]. Countries that were not rabies free were considered rabies-risk countries. Countries were classified based on the geographic regions, as defined by the United Nations [17].

### Inclusion and exclusion criteria

All records of imported infectious diseases reported from October 1, 2017, to October 31, 2019, were reviewed. Animal-related exposure was registered as one of the categories of imported infectious diseases in J-RIDA, and all cases with animal-related exposure were extracted for analysis. Japanese nationals with a confirmed diagnosis of animal-related exposure were included in the study. Patients with unconfirmed or foreign nationality, unknown pre-travel rabies vaccination status, and suspected pre-travel exposure were excluded from the study.

### Statistical analyses

Initially, we analyzed patient characteristics stratified by PrEP status. Next, we conducted a descriptive analysis of characteristics of animal exposure by different animal categories. A Kaplan–Meier curve was developed to estimate the time to from departure to animal exposure. Categorical variables are reported as frequencies and percentages. Continuous variables are reported as medians with interquartile ranges (IQR). Fisher’s exact test was performed to compare the types of animal exposure based on the type of animal. P values < 0.05 were considered statistically significant. All analyses were conducted using R software (version 3.5.1, R Core Team, 2018; R Foundation for Statistical Computing, Vienna, Austria). The survfit function in survival package was used for survival analysis.

### Ethical statements

This study was approved by the Ethics Committees of the National Center for Global Health and Medicine, Tokyo, Japan (approval number NCGM-G-002328-04) and of each participating hospital. The study was conducted in accordance with The Code of Ethics of the World Medical Association (Declaration of Helsinki). The patients’ data were anonymized before analysis, and the requirement of individual informed consent was waived because of the retrospective design of the study.

## Results

In total, 378 cases of animal exposure were retrieved. Forty-eight cases were excluded due to unconfirmed or foreign nationality, five cases were excluded due to unknown pre-travel rabies vaccination status, and three cases were excluded due to suspected pre-travel exposure. A total of 322 cases satisfied the inclusion criteria and were included in the analysis. Among 19 patients with PrEP, 12 had received three doses and seven had received one or two doses. The demographic characteristics of the patients with and without PrEP are shown in **Table 1**. Overall, 174 (54.0%) participants were men and 148 (46.0%) were women. The 20-to-29-years age group (n = 110, 34.2%) was the largest age group. There were 21 (6.5%) children under the age of 15 years.

**Table 1:** Demographic characteristics of patients according to pre-exposure rabies prophylaxis (N = 322).

Variables	Total (N = 322)	Non-PrEP (n = 303)	PrEP (n = 19)	p-value^a^
**Sex**				0.10
Male	174 (54%)	160 (52.8%)	14 (73.7%)	
Female	148 (46%)	143 (47.2%)	5 (26.3%)	
**Age (years)**				0.26
0–9	17 (5.3%)	17 (5.6%)	0 (0%)	
10–19	13 (4%)	13 (4.3%)	0 (0%)	
20–29	110 (34.2%)	100 (33%)	10 (52.6%)	
30–39	73 (22.7%)	66 (21.8%)	7 (36.8%)	
40–49	59 (18.3%)	58 (19.1%)	1 (5.3%)	
50–59	28 (8.7%)	27 (8.9%)	1 (5.3%)	
≥60	22 (6.8%)	22 (7.3%)	0 (0%)	
**Reason for travel**				0.47
Tourism in a non-package tour	176 (54.7%)	166 (54.8%)	10 (52.6%)	
Tourism in a package tour	69 (21.4%)	65 (21.5%)	4 (21.1%)	
Business	39 (12.1%)	37 (12.2%)	2 (10.5%)	
Visiting family or relatives	16 (5.0%)	16 (5.3%)	0 (0%)	
Education or student	11 (3.4%)	9 (3%)	2 (10.5%)	
Missionary, humanitarian or volunteer	6 (1.9%)	5 (1.7%)	1 (5.3%)	
Migration	2 (0.6%)	2 (0.7%)	0 (0%)	
Accompanying family members	1 (0.3%)	1 (0.3%)	0 (0%)	
Unconfirmed	2 (0.6%)	2 (0.7%)	0 (0%)	
**Travel period**				0.02
<2 weeks	213 (66.1%)	204 (67.3%)	9 (47.4%)	
2 weeks to 1 month	42 (13.0%)	40 (13.2%)	2 (10.5%)	
>1 month	30 (9.3%)	24 (7.9%)	6 (31.6%)	
Unconfirmed	37 (11.5%)	35 (11.6%)	2 (10.5%)	
**Number of countries visited**				0.128
1	283 (87.9%)	266 (87.8%)	17 (89.5%)	
2	19 (5.9%)	19 (6.3%)	0 (0%)	
3	12 (3.7%)	12 (4%)	0 (0%)	
≥4	8 (2.5%)	6 (2%)	2 (10.5%)	
**Pre-travel consultation**				<0.01
Yes	32 (9.9%)	21 (6.9%)	11 (57.9%)	
No	254 (78.9%)	249 (82.2%)	5 (26.3%)	
Unconfirmed	36 (11.2%)	33 (10.9%)	3 (15.8%)	
**Injured in countries with rabies risk**				0.25
Yes	286 (88.8%)	270 (89.1%)	16 (84.2%)	
No	14 (4.3%)	14 (4.6%)	0 (0%)	
Unconfirmed	22 (6.8%)	19 (6.3%)	3 (15.8%)	
**Outcome**				0.42
Outpatient	304 (94.4%)	286 (94.4%)	18 (94.7%)	
Referral to another medical facility	12 (3.7%)	12 (4%)	0 (0%)	
Inpatient	0 (0%)	0 (0%)	0 (0%)	
Unconfirmed	6 (1.9%)	5 (1.7%)	1 (5.3%)	

Data are presented as number (percentage). PrEP, pre-exposure prophylaxis

^a^P-values were calculated using the Fisher’s exact test.

The most frequent purpose for traveling was a tourism in a non-package tour (n = 175, 54.3%). In both the non-PrEP and PrEP groups, most trips were of a duration of less than 2 weeks, and most patients (n = 283, 87.9%) traveled to only one country. The number of pre-travel consultations was 32 (9.9%); among these, 11 (57.9%) patients did not have PrEP. The majority of the non-PrEP group were exposed in countries with rabies risk (n = 270, 89.1%). Most patients (n = 304, 94.4%) were treated as outpatients, and no patient required hospitalization. Twelve (3.7%) patients were referred to another medical facility.

Table 2 shows the destinations where the patients had animal exposures. We were unable to identify the destinations for 21 patients. In total, 254 (78.9%) patients traveled to Asia, including 180 (55.9%) to Southeast Asia, 38 (11.8%) to East Asia, and 27 (8.4%) to South Asia. The animals associated with the injuries were dogs (n = 177, 55%), cats (n = 82, 25.5%), monkeys (n = 50, 15.5%), and others (n = 13, 4%), including a tiger and other unspecified animals (Table 3). The majority of the exposure were classified into Category II or III (n = 70, 39.5% for Category II and 78, 44.1% for Category III). Dogs were likely to be owned, while cats and monkeys were likely to be stray or wild. The sites of injuries differed according to the animal type. Dogs tended to cause wounds to the legs (n = 99, 55.9%), whereas cats and monkeys were tended to cause wounds to the arms (n = 67, 81.7% of injuries by cats and n = 23, 46% of injuries by monkeys). Head and neck injuries were rare but were caused by monkeys (n = 7, 14%).

**Table 2:** Number of patients with animal exposure according to destination (N = 322).

Continent	Destination	Number of patients
Asia	Southeast Asia	180
	East Asia	38
	South Asia	27
	West Asia	7
	Central Asia	2
America	North America	10
	South America	9
	Central America	1
Africa	North Africa	9
	Sub-Saharan Africa	4
Europe	Western Europe	5
	Eastern Europe	3
Oceania		6
Unconfirmed		21

**Table 3:** Characteristics of animal exposure according to animal type (N = 322).

Variables	Dog (n = 177, 55%)	Cat (n = 82, 25.5%)	Monkey (n = 50, 15.5%)	Others^a^ (n = 13, 4%)	p-value^b^
**WHO category of contact with suspected rabid animals** ^**c**^					0.34
Category I	10 (5.6%)	5 (6.1%)	4 (8%)	3 (23.1%)	
Category II	70 (39.5%)	37 (45.1%)	17 (34%)	2 (15.4%)	
Category III	78 (44.1%)	35 (42.7%)	25 (50%)	7 (53.8%)	
Uncategorized	19 (10.7%)	5 (6.1%)	4 (8%)	1 (7.7%)	
**Exposure site**					<0.01
Lower extremities	99 (55.9%)	9 (11%)	13 (26%)	3 (23.1%)	
Upper extremities	57 (32.2%)	67 (81.7%)	23 (46%)	9 (69.2%)	
Trunk	12 (6.8%)	1 (1.2%)	6 (12%)	0 (0%)	
Head/face/neck	2 (1.1%)	1 (1.2%)	7 (14%)	0 (0%)	
Unknown	7 (4%)	4 (4.9%)	1 (2%)	1 (7.7%)	
**Owner status**					<0.01
Owned	68 (38.4%)	16 (19.5%)	3 (6%)	1 (7.7%)	
Not owned	78 (44.1%)	51 (62.2%)	42 (84%)	9 (69.2%)	
Unknown	31 (17.5%)	15 (18.3%)	5 (10%)	3 (23.1%)	
**Region of exposure**					<0.01
Southeast Asia	87 (49.2%)	48 (58.5%)	41 (82%)	4 (30.8%)	
East Asia	28 (15.8%)	9 (11%)	1 (2%)	0 (0%)	
South Asia	16 (9%)	3 (3.7%)	5 (10%)	3 (23.1%)	
West Asia	2 (1.1%)	5 (6.1%)	0 (0%)	0 (0%)	
Central Asia	1 (0.6%)	1 (1.2%)	0 (0%)	0 (0%)	
North America	6 (3.4%)	1 (1.2%)	0 (0%)	3 (23.1%)	
South America	9 (5.1%)	0 (0%)	0 (0%)	0 (0%)	
Central America	0 (0%)	0 (0%)	0 (0%)	1 (7.7%)	
North Africa	4 (2.3%)	4 (4.9%)	0 (0%)	1 (7.7%)	
Sub-Saharan Africa	2 (1.1%)	1 (1.2%)	0 (0%)	1 (7.7%)	
Western Europe	2 (1.1%)	3 (3.7%)	0 (0%)	0 (0%)	
Eastern Europe	3 (1.7%)	0 (0%)	0 (0%)	0 (0%)	
Oceania	5 (2.8%)	1 (1.2%)	0 (0%)	0 (0%)	
Unconfirmed	12 (6.8%)	6 (7.3%)	3 (6%)	0 (0%)	

WHO, World Health Organization

^a^Animals including tiger (n = 1) and unknown (n = 12).

^b^P-values were calculated using the Fisher’s exact test.

^c^Category I, touching or feeding animals, animal licks on intact skin (no exposure); Category II, nibbling of uncovered skin, minor scratches or abrasions without bleeding (exposure); and Category III, single or multiple transdermal bites or scratches, contamination of mucous membrane or broken skin with saliva from animal licks, exposure due to direct contact with bats (severe exposure).

**Fig 1:**
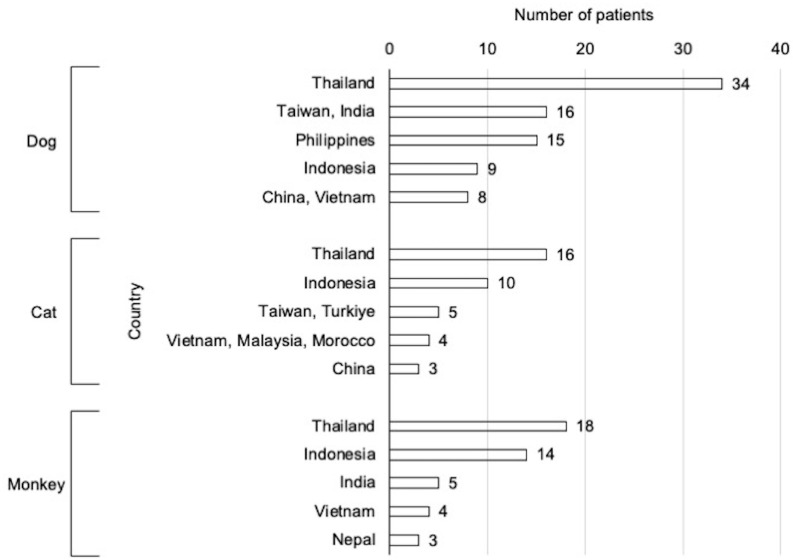
Countries where exposure occurred according to animal type. Countries with the highest number of patients exposed to dogs, cats, and monkeys are shown in descending order.

**Fig 2:**
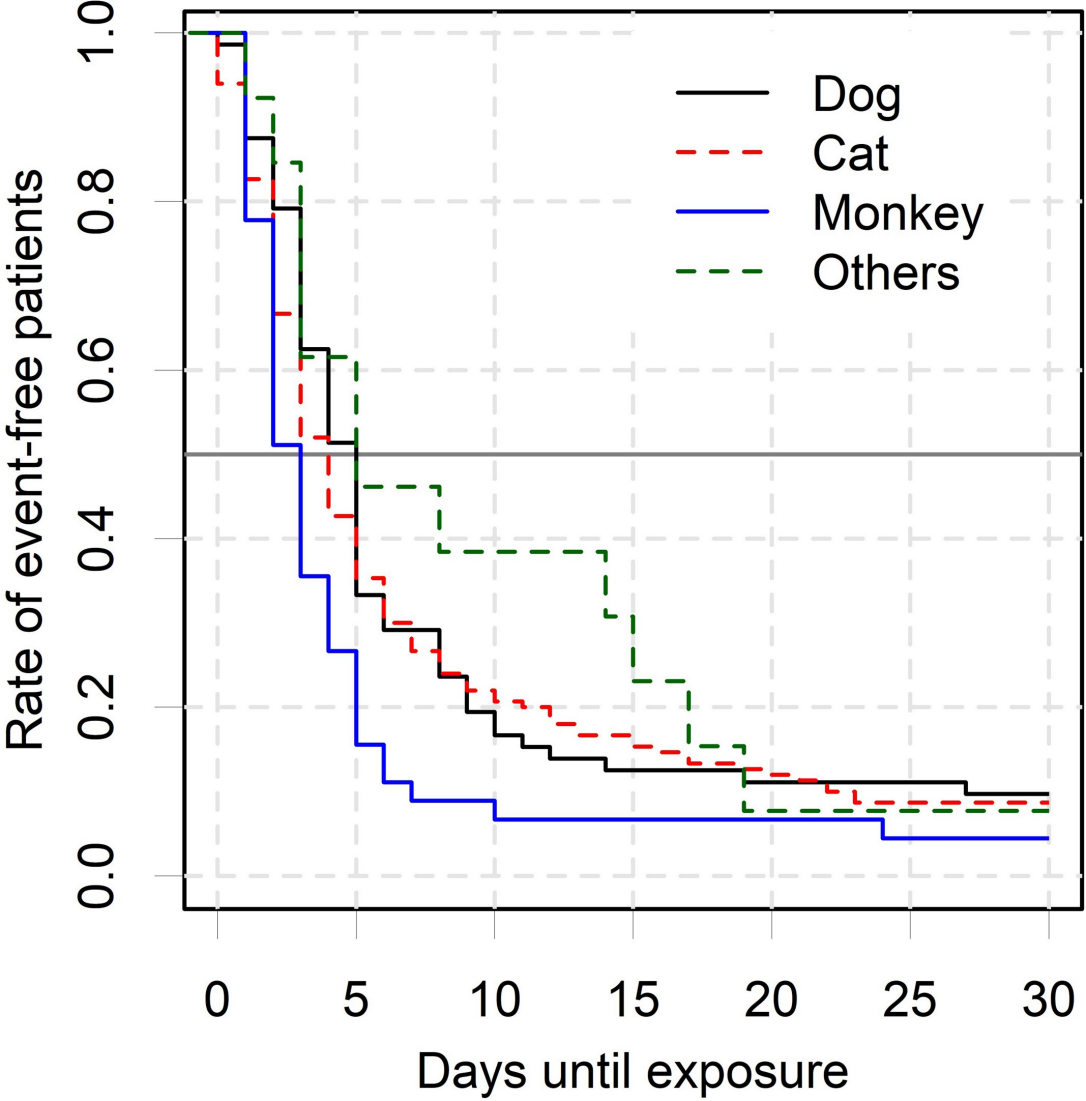
The Kaplan–Meier curve showing the proportion of exposure-free patients from time of departure according to animal type. Lines indicate dogs (solid black), cats (dotted red), monkeys (solid blue), and others (dotted green).

Fig 1 shows the common countries where patients were exposed to animals. Exposures commonly occurred in Southeast Asian countries, and Thailand had the highest frequency of exposure, regardless of the animal type. The median time to exposure was 5 days (IQR: 3–8 days) for dogs, 4 days (IQR: 2–8 days) for cats, and 3 days (IQR: 2–5 days) for monkeys. Regardless of the animal type, 50% of exposures occurred within 5 days from the start of travel (Fig 2).

## Discussion

To the best of our knowledge, this is the first study to evaluate potential rabies exposure among Japanese international travelers using multicenter registry data. Most Japanese patients were aged 20–30 years and traveled for non-package personal tours. GeoSentinel, a global clinician-based sentinel surveillance system for travel-related diseases [18, 19], reported that the median age of patients who had animal exposure while traveling abroad was 20–39 years, similar to that found in our present study. The proportion of children younger than 15 years was relatively smaller in our study than in other studies, which reported proportions ranging from 5.3–16.7% [20–23].

The pre-travel consultation rate in the present study might be much lower than the global rate. Previous studies suggested a low rate of pre-travel consultations in Japanese traveling in a rabies-endemic country. A questionnaire study for Japanese travelers to developing countries showed only 2.0% visited pre-travel clinics [24]. Another surveillance study conducted in Thailand [25] reported that 7.7% of Japanese travelers saw travel medicine specialists in advance [26]. Meanwhile, the GeoSentinel Surveillance Network reported a 30% pre-travel consultation rate among travelers [18]. The PrEP rate in developed countries has been reported to have a wide range of 1.7–16.0% [20–23, 27–30], suggesting the total PrEP rate in our study (5.9%) was not inferior to that in studies of other countries. Therefore, the problem for Japanese international travelers may be the lack of opportunity to learn about the dangers of animal exposure prior to travel, rather than a problem with PrEP vaccination rates. Many Japanese travelers may potentially be exposed to rabies because 89.0% of the countries where they acquired the injury were in risk areas for rabies in our study. Although the reasons that Japanese travelers have potential rabies exposures are unclear, a lack of qualified awareness of rabies has been reported to increase the risk of exposure for travelers [25, 26]. Most patients in our study were short-term travelers for tourism purposes. Our results might reflect their inadequate awareness of the dangers of overseas animal exposure.

In this study, the average duration of the trip was 1–2 weeks. Most patients visited only one country, and many of them were exposed to animals within 5 days of departure, suggesting that they tended to have animal exposure during short trips. Therefore, long duration of travel was not a useful indicator for rabies vaccination recommendations. A previous study has also suggested that long-term travel was not necessarily associated with high risk of rabies exposure [25]. Our findings suggest that international travelers could be at risk of rabies exposure during short-term travel to rabies-endemic countries. Knowledge about rabies and awareness are important to reduce the risk of animal exposure among travelers. Meanwhile, the cost and time required for vaccination are considered major underlying factors contributing to the non-administration of PrEP [31]. Currently, a two-dose PrEP, which is a simple, less expensive method of vaccination than a three-dose PrEP, is recommended by some institutions and has the potential to lead to PrEP dissemination for rabies [32, 33].

Regarding the types of animals that cause injury, dogs are the most likely cause of animal-related exposure in travelers, and more than half of the exposures in our study were caused by dogs. Dogs are a significant public health concern for rabies because 99% of rabies are caused by the exposure to dogs [15]. However, overseas travelers to Southeast Asia are also potentially at risk of exposure to rabies through contact with monkeys [23, 34]. Nonhuman primates, such as monkeys, are not primary rabies reservoirs and are uncommon cause for rabies. However, exposure caused by nonhuman primates is an important target for post-exposure prophylaxis (PEP) because rabies transmission from nonhuman primates to humans has been reported previously [35]. In our study, Thailand and Indonesia had the highest frequency of monkey exposure. In these countries, *Macaca fascicularis* are allowed to live in cities, the most famous of which is the Sacred Monkey Forest Sanctuary, colloquially known as “Monkey Forest,” in Ubud, Bali, Indonesia [34, 36]. Monkeys live close to human settlements to receive food from the people. The local monkey–human relationship provides an opportunity for monkey exposures among travelers.

Our study stratified the sites of exposure according to animal types. Notably, different animals have different modes of human-animal contact. Thus, it is important to consider whether animal exposure was caused by a provoked or an unprovoked event, while considering the active exposure prevention for travelers. Injuries caused by cats tended to be on the upper extremities, suggesting active human contact and that injuries caused by exposure to cats might have been provoked and could be prevented by humans modifying their behavior. Injuries caused by monkeys were observed on the face and upper and lower extremities.

This study has some limitations. First, despite the availability of information on the extent of animal exposure (WHO classification), there were no data on how the patients responded after animal exposure in each medical facility because J-RIDA did not include any information regarding the PEP and rabies immune globulin (RIG) the patients received. There was also no information on the treatment received in the destination country. Therefore, we were not able to examine the availability of PEP and RIG. The medical institutions participating in this study are major medical institutions in each region of Japan that treat travel-associated infectious diseases. This indicates that patients registered with J-RIDA were likely to have appropriately received rabies vaccines after exposure. Nevertheless, a previous study conducted in our institute found that the median time to first PEP was 4 days for Japanese patients exposed to animals overseas, suggesting that the time to access PEP in Japan may be delayed compared with that in other countries [12]. Meanwhile, RIG is not available in Japan, and travelers might not have access to RIG since its availability differs by country [37]. The study included a large number of patients with Category III injuries, many of whom may not have received PEP according to the WHO guidelines [11]. Second, it is difficult to evaluate the generalizability of this study because there is currently no information on the extent to which rabies PEP prophylaxis is covered at primary care clinics and hospitals that do not participate in J-RIDA. Future multicenter studies with large sample size on the status of PEP among Japanese travelers are warranted. Third, data on the number of PrEP doses prior to travel were collected in terms of three or fewer doses, and it is unclear how many people traveled with two doses of intramuscular PrEP, which is the dose currently recommended by the WHO and the US Centers for Disease Control and Prevention [11, 38]. This is because the J-RIDA registry was launched in 2017, before WHO’s recommendation for rabies PrEP was changed. Fourth, long-term international travelers or residents may complete PEP during their stay in other countries. These patients do not need PEP after returning to Japan, thereby leading to a low proportion of such patients in our study. Surveillance conducted in Thailand found more expatriates than travelers needed PEP after animal exposure [25], suggesting that long-term residents have more correct knowledge about rabies prevention and receive appropriate PEP in the country where the exposure occurred. Finding a way to disseminate knowledge of rabies to Japanese short-term international travelers is a challenge.

## Conclusions

Pre-travel consultation and awareness about rabies exposure through various overseas animals should be promoted among Japanese international travelers, including short-term travelers.
